# Exploring Microbial Diversity and Taxonomy Using SSU rRNA Hypervariable Tag Sequencing

**DOI:** 10.1371/journal.pgen.1000255

**Published:** 2008-11-21

**Authors:** Susan M. Huse, Les Dethlefsen, Julie A. Huber, David Mark Welch, David A. Relman, Mitchell L. Sogin

**Affiliations:** 1 Josephine Bay Paul Center for Comparative Molecular Biology and Evolution, Marine Biological Laboratory, Woods Hole, Massachusetts, United States of America; 2 Department of Microbiology and Immunology, Stanford School of Medicine, Stanford, California, United States of America; 3 Department of Medicine, Stanford University School of Medicine, Stanford, California, United States of America; 4 Veterans Affairs Palo Alto Health Care System, Palo Alto, California, United States of America; University of California Davis, United States of America

## Abstract

Massively parallel pyrosequencing of hypervariable regions from small subunit ribosomal RNA (SSU rRNA) genes can sample a microbial community two or three orders of magnitude more deeply per dollar and per hour than capillary sequencing of full-length SSU rRNA. As with full-length rRNA surveys, each sequence read is a tag surrogate for a single microbe. However, rather than assigning taxonomy by creating gene trees de novo that include all experimental sequences and certain reference taxa, we compare the hypervariable region tags to an extensive database of rRNA sequences and assign taxonomy based on the best match in a Global Alignment for Sequence Taxonomy (GAST) process. The resulting taxonomic census provides information on both composition and diversity of the microbial community. To determine the effectiveness of using only hypervariable region tags for assessing microbial community membership, we compared the taxonomy assigned to the V3 and V6 hypervariable regions with the taxonomy assigned to full-length SSU rRNA sequences isolated from both the human gut and a deep-sea hydrothermal vent. The hypervariable region tags and full-length rRNA sequences provided equivalent taxonomy and measures of relative abundance of microbial communities, even for tags up to 15% divergent from their nearest reference match. The greater sampling depth per dollar afforded by massively parallel pyrosequencing reveals many more members of the “rare biosphere” than does capillary sequencing of the full-length gene. In addition, tag sequencing eliminates cloning bias and the sequences are short enough to be completely sequenced in a single read, maximizing the number of organisms sampled in a run while minimizing chimera formation. This technique allows the cost-effective exploration of changes in microbial community structure, including the rare biosphere, over space and time and can be applied immediately to initiatives, such as the Human Microbiome Project.

## Author Summary

Microbes play a critical role in both human and environmental health. The more we explore microbial populations, the more complexity and diversity we find. Phylogenetic trees based on 16S ribosomal RNA genes have been used with great success to identify microbial taxonomy from DNA alone. New DNA sequencing technologies, such as massively parallel pyrosequencing, can provide orders of magnitude more DNA sequences than ever before, however, the sequences are much shorter, so new methods are necessary to identify the microbes from short DNA tags. We demonstrate the effectiveness of identifying microbial taxa by comparing short tags from 16S hypervariable regions against a large database of known 16S genes. Using this technique, hypervariable region tags provide equivalent taxonomy and relative abundances of microbial communities as full-length rRNA sequences. The greater sampling depth afforded by tag pyrosequencing uncovers not only the dominant microbial species, but many more members of the “rare biosphere” than does capillary sequencing of the full-length gene. Tag pyrosequencing greatly enhances projects exploring composition, diversity, and distribution of microbial populations, such as the Human Microbiome Initiative. A companion paper in *PLoS Biology* (see Dethlefsen et al., doi:10.1371/journal.pbio.0060280) successfully uses this technique to characterize the effects of antibiotics on the human gut microbiota.

## Introduction

The biosphere contains between 10^30^ and 10^31^ microbial genomes, at least 2–3 orders of magnitude more than the number of plant and animal cells combined [1]. Microbes control global utilization of nitrogen through nitrogen fixation, nitrification, and nitrate reduction, and drive the bulk of sulfur, iron and manganese biogeochemical cycles [2]. They regulate the composition of the atmosphere, influence climates, recycle nutrients, and decompose pollutants. Without microbes, multi-cellular life on earth would not have evolved and biology as we know it would not be sustainable.

The diversity of microbial communities and their ecologic and metabolic functions are being explored across a great range of natural environments: in soils [3]–[5], air [6] and seas [7]–[10], on plants [11] and in animals [12],[13] and in extreme environments such as the arctic [14], deep-sea vents [15], uranium-contaminated soil [16], and waste-water treatment discharge [17]. In recognition of the role marine microbes play in the biogeochemical processes that are critical to life in all environments on Earth including carbon and nitrogen cycling, the International Census of Marine Microbes (ICoMM: http://icomm.mbl.edu) has launched an international effort to catalogue the diversity of microbial populations in the oceanic, coastal, and benthic waters. Microbes associated with human health will be intensely studied through two recent large-scale initiatives: the Human Microbiome Project sponsored by the NIH (http://nihroadmap.nih.gov/hmp/) and MetaHIT sponsored by the EU (http://www.metahit.eu), which seek to characterize the composition, diversity and distribution of human-associated microbial communities. Other recent human health studies include microbes in breast milk [18], chronic wounds [19], human gut [20], dental caries [21], and childcare facilities [22].

Microbes associated with the human body outnumber human cells by at least a factor of ten [23]. Some microbes cause disease, but the overwhelming majority are either innocuous or play a role in human physiology, including immune response, digestion and vitamin production. As recently as the late 1980's, descriptions of human-associated microbiota were constrained by cultivation technologies. Over the last twenty years, sequencing surveys of amplified regions of small subunit ribosomal RNA (SSU rRNA) genes have revealed that microbial diversity is much greater than the 5,000 microbial species described using phenotypic features in Bergey's taxonomic outline [24], and that microbial communities are far more complex than initially thought. For instance, *E. coli*, once thought to be a dominant species in the human gut, is clearly a minor member relative to various members of the phyla *Bacteroidetes* and *Firmicutes*. It is now evident that microbiologists have been successful in culturing fewer than one percent of the different kinds of single cell organisms from most microbial communities [25]. Even for well-studied communities, such as the human distal gut, only 20–40% of the microbes have been cultured. Deeper surveys with new approaches are revealing ever-greater diversity. Even these studies, with hundreds of thousands of microbes sampled, have not been extensive enough to provide a complete picture of the diversity (richness) and relative abundance (evenness) of microbial communities.

To explore these questions, microbiologists must be able to compare microbial communities within and across individuals, in different states of health and disease, and over time. The first step in these community analyses is to develop detailed descriptions of each population, including low abundance taxa that comprise the rare biosphere [9]. Exploration of the human microbiome can leverage methods used to explore the microbiome of other environments such as soil, the deep sea, and other vertebrate microbiomes.

By necessity, microbiologists have historically focused their efforts on the dominant components of microbial communities. Recognizing the importance of gathering information about high, medium and very low abundance taxa, Sogin et al. [9] introduced the use of massively-parallel DNA sequencing of short hypervariable regions of SSU rRNA to characterize microbial populations. In a subsequent study that collected nearly one million short hypervariable region tags, Huber et al. [15] demonstrated that there are over ∼40,000 different kinds of bacteria and archaea in a few liters of hydrothermal vent fluid. Rarefaction data from this study and others show that in many environments, even this level of sequencing is insufficient to fully describe microbial diversity [5],[26].

The lower cost and higher throughput of pyrosequencing employed in these studies allows for sampling efforts that are orders of magnitude greater than traditional capillary dideoxy sequencing of cloned SSU rRNA amplicons [27]. With the recently announced capability to sequence >400 nt, it will be possible to span most hypervariable regions, multiple adjacent hypervariable regions, or possibly combinations of non-adjacent hypervariable regions through paired-end sequencing strategies. However, comparisons of 400 nt reads from rapidly evolving rRNA regions do not contain sufficient evolutionary information to infer robust phylogenetic relationships and the hundreds of thousands of reads produced in a single experiment far exceeds the limitations of current phylogenetic software. Tags from the V6 region are also too short for current implementations of Bayesian classifiers such as the Ribosomal Database Project Classifier (RDP) [28]. However, each read represents a hypervariable region tag of an SSU rRNA gene present in the sample. We developed a tag search engine, Global Alignment for Sequence Taxonomy [9], GAST, which utilizes existing databases of full-length SSU rRNA genes and their pre-computed phylogeny for high-throughput taxonomic analysis of microbial communities using hypervariable region tag sequences.

The use of a single, small hypervariable region tag for assigning taxonomy presents several challenges. The information content in a short hypervariable region sequence may not be sufficient for inferring taxonomic affinity. BLAST [29] alone is insufficient to consistently identify the sequences in molecular databases that are the closest matches to tag queries along their full length. Here we analyze the reliability of assigning taxonomic identifiers based solely on tags, specifically using the V3 and the V6 hypervariable regions of SSU rRNA. Using SSU rRNA genes from the human gut and deep-sea vents, we compare the taxonomic assignments of the full-length sequences with the taxonomic assignments of their V3 and V6 regions, excised in silico. We then examine microbial populations of the human gut in greater detail, using both massively-parallel pyrosequencing of hypervariable region tag and Sanger-generated full-length sequences, to determine if any differences in sampling and taxonomic assignment exist with these two sequencing strategies. In a companion paper [30], we used GAST to examine the impact of the antibiotic ciprofloxacin on population structures of the human microbiome.

## Results

### Assessment of Hypervariable Region Specificity in the RefSSU Database

Tags from a hypervariable region must map to a full-length SSU rRNA with minimal ambiguity to serve as reliable phylogenetic markers; i.e. phylogenetically distinct lineages should not contain identical tags. Most of the redundant SSU rRNA sequences containing identical sequences for a particular hypervariable region in the database are from the same genus. This redundancy does not interfere with the use of the hypervariable region tags for taxonomy, but rather strengthens the assignment. For each unique V3 and V6 sequence, we examined the number and taxonomy of all the source full-length sequences. We treat each hypervariable region independently, since two full-length rRNA sequences with identical V3 regions may differ at the V6 region or another hypervariable region. Of the 59,830 unique V6 reference sequences, 74% mapped to one SSU rRNA sequence, 10% mapped to 2 sequences, and only 5% mapped to 7 or more. The V3 region, which is longer, showed slightly better resolution: 82% mapped to one SSU rRNA, 8% mapped to 2 sequences, and only 3% mapped to 7 or more (results not shown). Although only a small percentage of tags map to more than a few SSU rRNA sequences, this included some that mapped to a very large number of different SSU rRNA sequences. For example, 3 V6 reference sequences and 8 V3 reference sequences each mapped to more than 1000 different SSU rRNA sequences.

In almost all cases where a hypervariable tag sequence maps to more than one full-length SSU rRNA sequence, the overwhelming majority of full-length sequences still map to only one genus (Table 1). Since RefSSU sequences that give rise to identical hypervariable region tags are generally from the same or highly similar organisms, V6 and V3 tags can be unambiguously mapped to the genus level 97% and 99% of the time, respectively. Even if we examine only the subset of V3 sequences that mapped to multiple SSU rRNA sequences, 95% map uniquely to genus, 98% to family, and 99% to order, class and phylum. Similarly for the V6 region, 91% of tags derived from multiple SSU rRNA entries map uniquely to genus, 96% to family, 97% to order and 99% to class and phylum. Even in those cases where reference tags mapped to more than 1,000 full-length sequences, in 9 of the 11 cases there was one dominant taxon. Of the other two cases, one had complete consensus to the order level and the other had all but two matching at family level. Not only do most of the reference tags in the database have only one SSU rRNA source, even those from multiple SSU rRNA sources still represent almost exclusively one taxon.

**Table 1:** Percent of hypervariable region tags from the RefSSU database that map to one or more taxa.

Hypervariable region V3					
Number of Taxa	1	2	3	4	5+
**Phylum**	99.96% / 114328	0.04% / 42	0.00% / 42	0	0
**Class**	99.93% / 109352	0.07% / 77	0.00% / 2	0	0
**Order**	99.88% / 99682	0.12% / 113	0.00% / 4	0	0
**Family**	99.62% / 88015	0.34% / 297	0.04% / 34	0.00% / 3	0.00% / 3
**Genus**	99.11% / 69686	0.07% / 495	0.09% / 64	0.05% / 35	0.00% / 29
**Hypervariable region V6**					
**Number of Taxa**	**1**	**2**	**3**	**4**	**5+**
**Phylum**	99.83% / 54728	0.17% / 94	<0.01% / 1	0	0
**Class**	99.68% / 51795	0.30% / 158	0.01% / 6	0	0
**Order**	99.30% / 45579	0.62% / 285	0.05% / 23	0.01% / 5	0.01% / 6
**Family**	98.77% / 40454	1.08% / 444	0.11% / 45	0.03% / 11	0.01% / 5
**Genus**	97.33% / 31463	2.07% / 670	0.35% / 112	0.15% / 50	0.01% / 31

For GAST to accurately identify microbial taxa, the same tag sequence must not be present in different taxa: each tag should only have one taxonomic source. Table 1 shows the percentage of hypervariable tags (V3 or V6) present in the RefSSU that map to single or multiple taxa. The results are displayed both as a percentage of tags and as a total number of tags.

### Comparison of Taxonomy from Long SSU rRNA Sequences vs. In Silico–Generated Hypervariable Region Tags in the Human Gut and Deep-Sea Vent Microbiomes

**Figure 1:**
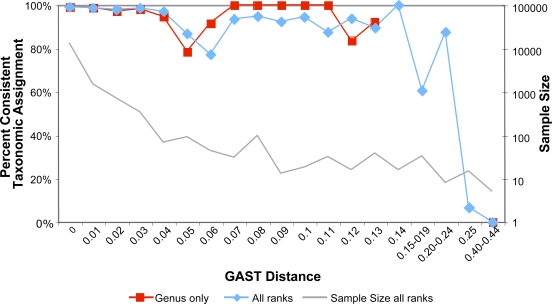
Accuracy of GAST taxonomic assignments at increasing distances from the reference database. The percent of hypervariable region tags mapping to the same taxonomy as their source full-length SSU rRNA sequences is plotted as a function of the distance of the tags from their nearest GAST match in the reference database. Data from both the human gut and deep-sea vent for both hypervariable regions (V3 and V6) were combined to provide enough data points at greater distances. Only data points represented by a minimum of 5 tags were included. The GAST process consistently maps tags to the same taxonomy as their source for GAST distances up to 0.15 – a 15% divergence of the tag from its nearest match. Our combined datasets include only 61 tags (less than 0.4% of our tags) with a GAST distance > = 0.15, insufficient data to analyze the accuracy of the GAST process for these larger distances.

We compared the taxonomy of full-length SSU rRNA sequences assigned by RDP to the taxonomy of both their V3 and V6 regions (generated in silico from full-length rRNA SSU sequences) as assigned by GAST. We used two independent datasets of full-length sequences: 7215 sequences from the human gut [30] and 1058 sequences from deep-sea vents. The taxonomy assigned by GAST to either the V3 or V6 hypervariable regions from the human microbiome and vent datasets matched the RDP taxonomy of the parent SSU rRNA sequences at the genus level 99+% and 98% of the time, respectively (Table 2). Both datasets provided agreement between hypervariable region tags and full-length sequences in more than 96% of instances: the human microbiome data were classified consistently in 99% or more instances. The human microbiome tags were better represented in the reference database than were those from deep-sea vents. For the human tags, 91% were exact matches to a reference tag and 99.7% of them were within 10% similarity of the nearest reference tag. Only 51% of the deep-sea vent tags had an exact match in the reference database and only 90% were within 10% similarity of the nearest match. Despite this greater divergence from the reference sequences, the GAST process still credibly mapped the taxonomy of the deep-sea vent tags. Figure 1 shows the level of agreement between the tags and full-length sequences as a function of GAST distance. The GAST process consistently provides an accurate mapping of taxonomy for tags approaching a 15% divergence from their nearest match in the reference database. For tag and full-length source pairs where both mapped to the genus level, the average fidelity of assignment is greater than for pairs where one or both could not be mapped as far. We have insufficient data for tags greater than 15% divergence (less than 0.4% of the data points) to adequately analyze the accuracy of taxonomy for these greater GAST distances. We expect that beyond some threshold value greater than or equal to 15%, the accuracy will decrease with increasing GAST distances.

**Table 2:** Comparison of taxonomic assignments using full-length and in silico generated V3 and V6 hypervariable region tags.

	Human Gut Microbiome V3 region			Deep-sea Vents Microbiome V3 region		
	Count	Same	%Same	Count	Same	%Same
**Superkingdom**	7208	7208	100.00% / 100.00%	963	963	100.00% / 100.00%
**Phylum**	7168	7145	99.68% / 99.82%	920	901	97.93% / 97.68%
**Class**	7033	7002	99.56% / 99.52%	884	857	96.95% / 99.55%
**Order**	7019	6987	99.54% / 99.57%	807	784	97.15% / 100.00%
**Family**	5726	5688	99.34% / 98.93%	764	746	97.64% / 98.44%
**Genus**	5178	5153	99.52% / 97.99%	701	686	97.86% / 99.10%
	**Human Gut Microbiome V6 region**			**Deep-sea Vents Microbiome V6 region**		
	**Count**	**Same**	**%Same**	**Count**	**Same**	**%Same**
**Superkingdom**	7215	7215	100.00%/ 100.00%	1058	1058	100.00% / 99.53%
**Phylum**	7175	7152	99.68% / 99.82%	1008	986	97.82% / 90.00%
**Class**	7040	7009	99.56% / 99.52%	970	939	96.80% / 90.08%%
**Order**	7026	6994	99.54% / 99.56%	881	847	96.14% / 87.06%
**Family**	5731	5693	99.34% / 98.93%	833	814	97.72% / 87.23%
**Genus**	5183	5158	99.52% / 97.97%	766	749	97.78% / 88.52%

Treating the V3 and the V6 regions independently, we counted the number of assignments the GAST process made at each taxonomic rank and the number and percent of times those assignments were the same as the assignment given to the full-length source sequence. The second percent value is the rate at which the top BLAST match predicted the same assignment as the full-length source.

We compared the use of GAST to assign taxonomy with the use of the top BLAST match (Table 2). While the top BLAST match was consistent with GAST to the order level, it was less accurate for family and genus for the V6 region, especially for the deep-sea vent sequences. In addition, the top BLAST match was often not the best GAST match in cases where the taxonomic assignment was the same (Table 3). A comparison of the BLAST rank vs. GAST distance did not show any significant correlation (results not shown).

**Table 3:** BLAST ranks for top GAST hits.

	Human Microbiome		Deep-Sea Vents	
BLAST Rank	V3 region	V6 region	V3 region	V6 region
**1**	83.70%	94.90%	75.17%	81.89%
**2**	13.48%	4.34%	5.27%	7.16%
**>2**	2.82%	0.76%	19.57%	10.95%

The percentages reported represent the frequency with which the top GAST match corresponds to the top BLAST match, the second best BLAST match, or any other BLAST match.

### Comparison of Population Sampling Using Sanger-Generated Full-Length and Pyrosequencing-Generated V3 and V6 Tags for the Human Gut Microbiome Dataset

**Figure 2:**
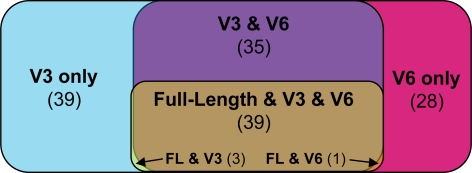
Correspondence of genera found by each method. The Venn Diagram shows the extent of overlap between the V3, V6 and full-length sequencing of the human gut microbiome. The V3 tag sequencing found the most genera (116), the V6 found 103 genera, and full-length sequencing found only 43 genera.

We compared taxonomic assignments and their frequencies for the human gut microbiome data sampled with full-length SSU rRNA (n = 7215, length = 1300–1450 nt), V3 tags (n = 422,992, trimmed length = 100–200 nt) and V6 tags (n = 441,894, trimmed length = 50–70 nt) [30]. Full-length sampling detected a total of 43 genera, V3 pyrosequencing detected 116 genera and V6 pyrosequencing detected 103 genera (Figure 2). V3 sampling detected 74 genera that were not detected using the full-length sequencing and V6 sampling detected 60 genera (102 different genera combined). No genera were detected by the full-length sequencing alone. V3 sampling missed one taxon represented in the full-length sequences: Escherichia, detected 5 times with the full-length and once with the V6. V6 sampling missed three taxa, Hespellia, Klebsiella, and TM7 detected by the full-length with only one sequence each (detected 12, 16 and 4 times with V3, respectively). Only the TM7 sequences could be affected by a primer bias; the other two taxa have exact matches to forward and reverse primers as represented in the reference database of full-length SSU rRNA. The TM7 includes several different primer region sequences in the reference database each of which is one or two bases from its nearest primer, but only at the 5′ end, which should still detect abundant genera. The lack of TM7 in the V6 pyrosequencing data could be caused by a primer bias acting on a rare population, or could simply be the undersampling of rare organisms. In sum, less than 1% of the taxa identified by full-length sequences, representing <0.1% of the sequences, were missed with the V3 and less than 3% were missed by the V6, representing <0.5% of the sequences. Full-length sequencing, on the other hand, missed 73 of the 116 of the genera identified by V3 and 60 of the 103 of the genera identified by V6.

A comparison of the relative effectiveness of the two hypervariable regions showed only minor differences. The taxa represented in one variable region dataset and not the other (42 found only in V3, 26 found only in V6) were all of very low abundance, with only 3 taxa occurring more than 20 times. V3 tags identified 21 *Lonepinella* and 34 *Megasphaera* that were not identified by the V6 tags. V6 tags identified 46 *Paenibacillus* that were not identified by the V3 tags. Of these three taxa, only *Megasphaera* could have been caused by a primer bias, there being a one-base mismatch in the forward primer region. The other two genera had perfect matches to both primers. Each of the classes and phyla discovered by only one hypervariable region were represented by fewer than 10 sequences. Only two orders or families containing more than 10 sequences (11 *Chromatiales Chromatiaceae*, and 16 *Acholeplasmatales Acholeplasmataceae*) were present in only one sample set (V6) both of which have exact matches to our V3 primers.

**Figure 3:**
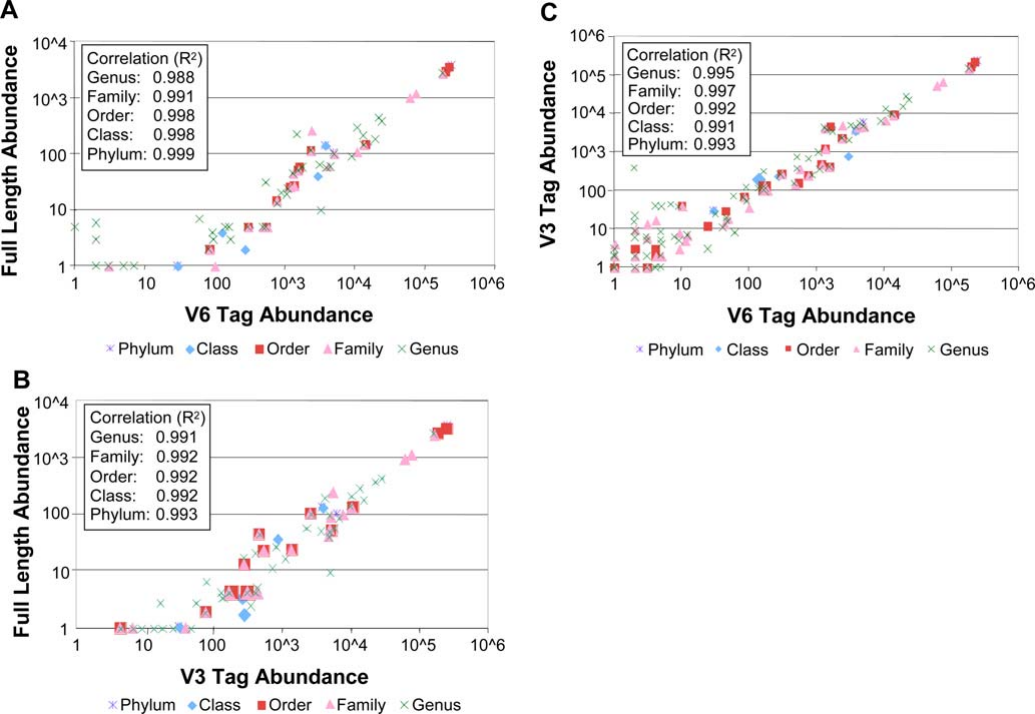
Correlation of taxonomic assignments for human gut sequences based on full-length SSU rRNA, V3 and V6 variable regions. At each taxonomic level, the number of sequences from a particular taxon using one sequencing strategy (e.g., V6 tags) is plotted against the number of sequences from that same taxon using a second sequencing strategy (e.g., full-length SSU rRNA genes). For instance, classifying to the genus level, the Clostridial genus *Ruminococcus* occurred 186 times in the full-length SSU rRNA sequences and 19,332 times in the V6 tags. The order Clostridiales occurred 3,613 times in the full-length sequences, and 217,482 times in the V6 tags. *Note*: correlations are linear, although the axes use log scales for clarity. Figure 3A compares V6 tags with full-length sequences, Figure 3B compares V3 tags with full-length sequences, and Figure 3C compares V6 and V3 tags.

**Figure 4:**
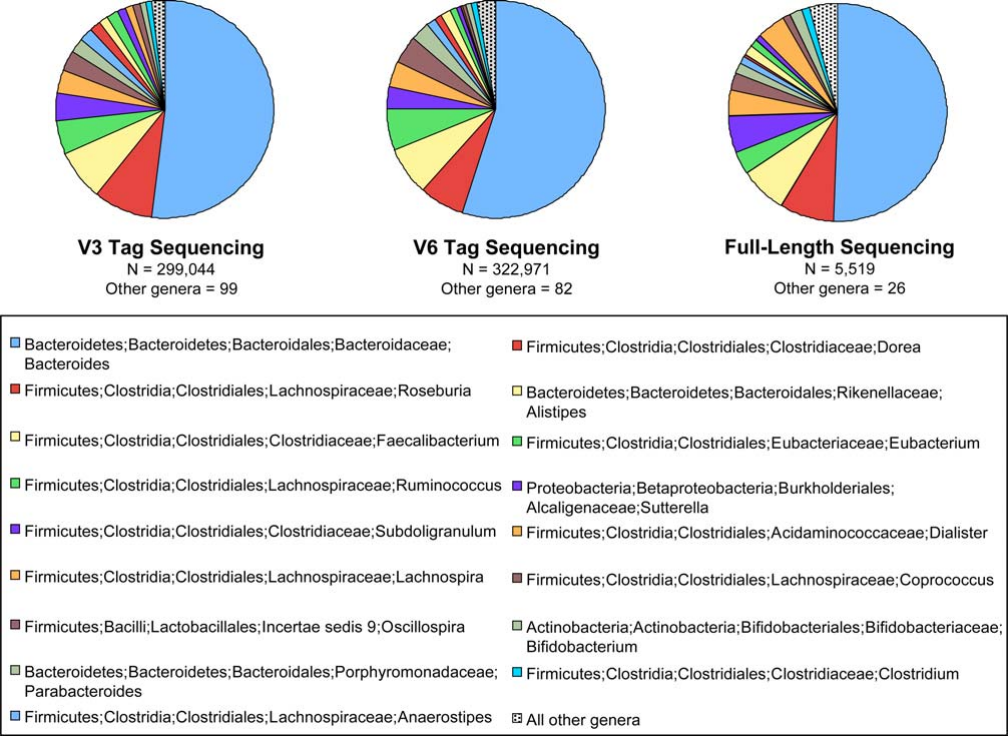
Comparison of taxonomic assignments for human gut sequences at the genus level. Assignment to genus and their relative abundances are distinctly similar for each of the three methods, V3 tag, V6 tag and full-length sequencing of SSU rRNA genes. Only sequences classified to the genus level are included. The tag sequencing approach, however, reveals many more rare taxa than does conventional full-length sequencing.

The frequencies of each taxonomic assignment at each level (phylum, class, order, family, and genus) show a linear correlation between the full-length sequencing and each of the hypervariable region tag sequencing sets with an R^2^ correlation value of 0.99 (Figures 3A and 3B). Taxonomic abundance levels revealed by each of the two hypervariable region tag sequencing datasets also correlated strongly with each other (R^2^ = 0.99) at all taxa levels (Figure 3C). The x-intercepts of the data in Figures 3A and 3B show the level to which uncommon taxa were sampled by tag sequencing but missed by full-length sequencing. Figure 4 visually compares the relative abundances of the dominant taxa in each sample, and the numbers of rare taxa detected by each.

## Discussion

The goal of fully describing microbial diversity in the natural world, including the human microbiome, remains elusive. The challenge posed by the unseen species is analogous to the ‘dark matter’ problem in astrophysics and is made more difficult by the unevenness of natural communities, especially in the intestinal tract of animals, and the uncertainty about the potential importance of rare species. New surveying tools are needed to provide the breadth and depth of sampling necessary to study the behavior of microbial populations and the rare species within them. Towards this aim, the low cost per read and high throughput of massively parallel pyrosequencing provide a means for deeply sampling these environments. Although the read length of pyrosequencing is increasing, it is unlikely that the technology will produce hundreds of thousands of full-length rRNA sequences per run in the near future. While this and other next generation sequencing technologies may not be appropriate for generating full-length SSU rRNA sequences used in traditional analyses, they are particularly well suited for a tag sequencing strategy, where each read represents a short amplicon that can be used as a tag to match the sequence to known full-length sequences.

Microbial ecology requires not only identification of organisms, but also consistent and reproducible sampling of populations. We sampled the human gut microbiota with conventional long SSU rRNA sequences and with V3 and V6 tag sequencing, comparing both the types of organisms and their relative abundances (Figures 2, 3, and 4). At all taxonomic ranks and for both hypervariable regions, the composition of the microbiota as revealed using long sequences or tag sequences correlated for 99% of the taxa. These results applied to all taxonomic ranks (genus to phylum) and to both hypervariable regions (V3 and V6). The greater sampling depth provided by the massively-parallel pyrosequencing of hypervariable tags provides a greater window onto the breadth of taxonomy in the human gut than the use of longer full-length sequences. This is illustrated by the large x-intercept of the data in Figures 3A and 3B. Each set of tag sequences showed measurable quantities of taxa (x>1) that were lost in the full-length sequencing (y = 0). The x-intercept comparing the two tag sequencing experiments (Figure 3C) is at the origin, implying that the two hypervariable regions were comparable in elucidating the rare biosphere. Primer bias was shown to be negligible in most cases, and undersampling is the most likely cause for the differences in small populations detected. Both tag sequencing experiments found similar numbers of taxa and substantially more than in the full-length sequencing.

For tag sequencing of hypervariable regions to be effective for mapping taxonomy, specific sequences must match unambiguously to source organisms. If it were common for two divergent organisms to have the same or highly similar V3 or V6 regions, tag sequencing would not be an accurate means for assigning taxonomy. In our reference database of over 500,000 rRNA sequences, we found that hypervariable region tags map to individual taxa with high fidelity. In only a few cases for either the V3 or the V6 region did we find sequences that exactly match two or more distinct taxa (Table 1). The reference databases are replete with highly studied bacteria, and multiple copies of a hypervariable region for these organisms do not imply any taxonomic ambiguity. The V3 region was 99% accurate to the genus level. The V6 region was only slightly less resolved than the V3, still providing a 97% accuracy in assignment to the genus level, 98+% accuracy to the level of family, and 99% accuracy at the level of order. The V3 region is longer than the V6, which should have a positive effect on its specificity. The RefV6 database contains about half the number of reference sequences as the RefV3, and the accuracy of the V6 may be even greater as the reference databases grow. These levels of accuracy show that hypervariable region tags contain adequate information to accurately map taxonomy of both bacterial and archaeal organisms.

To assign taxonomy to our long SSU rRNA gene sequences, both in our RefSSU database and in our experimental sequences, we used the Ribosomal Database Project classifier (RDP). RDP is not 100% accurate and some of the ambiguities in the reference database could be attributable to limitations with the RDP classifier. For tag sequencing with the GAST process, however, we are assessing the utility of a tag as a surrogate for the longer SSU rRNA sequences via a look-up and distance matrix. Can we consistently assign the correct taxonomy to both a SSU rRNA sequence and its constituent hypervariable regions independently? The RDP taxonomy provides a consistent and high-quality taxonomic classification (Bergey's taxonomy), facilitating our analysis. Slight inaccuracies in RDP are not an important factor in whether a tag sequence can be used as a surrogate for a full-length SSU rRNA sequence.

We conducted an in silico experiment assessing taxonomic assignment using tags of hypervariable regions extracted from full-length sequences and compared the tags directly to the full-length sequences. We used two independent datasets, a human gut microbiome SSU rRNA dataset, which should be relatively well represented in the reference databases of SSU rRNA genes, and one of deep-sea vent microbes, which are less well studied and therefore less well represented in the reference databases. We examined the use of both the V3 and the V6 region tags to assign taxonomy for both groups of microbes. In all four cases, we found excellent correspondence between the use of the GAST process for assigning taxonomy to short hypervariable region tags and the use of RDP for assigning taxonomy to the full-length sequences. For both variable regions from the human microbiota, the taxonomic assignments of the tags agreed with the long sequences at a rate consistently greater than 99%. The deep-sea vent data agreed in over 97% of instances at the genus level.

The two variable regions mapped taxonomy with virtually identical fidelity. The greater difference was not in choice of variable region, but in the environment examined. Of the human gut microbes sampled, 91% of all tags had exact matches in the reference database and virtually all had matches within a 10% sequence match. Of the deep-sea vent microbes, which have not historically been as well studied, only 51% had exact matches in the reference database and only 90% were within a 10% sequence match of a reference sequence. Despite the fact that as many as 10% of the deep-sea vent tags did not have a close match in the reference database, the GAST process only mis-assigned 3% of the tags. The GAST process accurately assigns taxonomy to tags diverging as much as 15% from their nearest reference match (Figure 1). Although our data included insufficient tags with GAST distances greater than 0.15 to fully assess their accuracy, the ability to transfer taxonomic information from the reference database is will certainly be less accurate at greater GAST distances. The deep-sea vent mismatch rate was similar for both hypervariable regions and at each taxonomic level from genus to phylum, and less than one-third of the mismatches were for tags >15% divergent. A possible explanation is the deep-sea vent environment may contain phyla that are not yet adequately described, and whose full-length SSU rRNA sequences are therefore not well classified by the RDP. These results imply that hypervariable region tag sequencing and the GAST process are excellent tools for assigning taxonomy, but they cannot overcome basic gaps in knowledge of the under-explored areas of the microbiome. As more is learned about these organisms, and their full-length SSU rRNA genes are added to the reference databases, hypervariable region tags sequencing projects will directly benefit, and taxonomy will improve.

The top BLAST matches for the human gut microbiome mapped taxonomy better than the top BLAST matches for the deep-sea vents. This is likely because the human microbiome data are better represented in the reference database. Since 91% of the human microbiome tags had exact matches in the reference database these should be consistently identified by BLAST as the best match. For the deep-sea samples where only 51% had exact matches, a top BLAST hit may find only a local match within the sequence rather than a globally-weighted match as with GAST. Reliance on local alignments can be misleading: 80% of the deep-sea vent V6 tags that did not rank among the top two BLAST hits showed a BLAST alignment length shorter than the tag sequence for the top BLAST hit. BLAST failed to identify the correct genus for 11% of these V6 tag sequences, whereas GAST which failed for only 2%. An increase in distance from the tag to the nearest reference sequence using GAST did not correlate with a lower BLAST rank. The magnitude of divergence from the reference database does not explain the difference between V3 and V6 regions. The V3 tags were noticeably more divergent from their top BLAST hit than were the V6 tags, despite the larger dataset of reference V3 sequences. This did not adversely affect the ability to identify tags to the genus level. Since the RDP taxonomy is restricted to the genus level, we could not review the BLAST ranks for species-level.

Full-length sequencing missed 63% of the genera identified by V3 and 58% of the genera identified by V6 (for more details, see Dethlefsen *et al.* [30]). The full-length sequencing uncovered only four rare taxa missed by one or the other hypervariable region, but no taxa missed by both of the hypervariable regions. Primer mismatches were minimal, but may be relevant when combined with a very low abundance. The hypervariable region tag sequencing did not introduce any strong biases against the discovery of common taxa or the relative abundance of these taxa in this experiment. As predicted, the hypervariable region tag sequencing provided a much greater breadth and depth of sampling.

Although the level of sampling with tag sequencing is orders of magnitude greater than with traditional methods, a single pyrosequencing run (with >400,000 sequences) is still insufficient to fully sample the rare biota in the human distal gut. The sampling limitation of this experiment can be seen in the small but distinct number of taxa that appeared in one but not the other tag sequencing experiment. All were of low abundance and are dispersed throughout the microbial world rather than clustering in one specific taxon. Since no common taxa were omitted by sequencing of either hypervariable region, any effects of primer bias are limited to rare taxa and cannot be discerned from the effects of undersampling.

Other methods such as Greengenes [31] and SeqMatch [32] use short tag sequences to determine phylogenetic affinity through comparisons to reference data sets of nearly full-length SSU rRNA sequences without requiring the inference of phylogenetic trees from the hypervariable regions. Greengenes (http://greengenes.lbl.gov) uses NAST [33] to align tags by inserting them into a pre-existing database of >10,000 aligned full-length sequences, and then assigns taxonomy based on the nearest neighbor in the database. Liu et al [34] used NAST on simulated tag sequences and found similar Unifrac clustering results from the tags as from their full-length source sequences. The Greengenes website is limited to 500 sequences and uses a database of only 10,000 sequences for comparison and does not perform a consensus of multiple taxonomy matches.

SeqMatch uses a k-nearest-neighbor, word-matching algorithm rather than a multiple sequence alignment to display nearest matches in the RDP dataset, and uses the lowest common taxon for consensus (essentially a unanimous consensus rather than 66% used by GAST). The RDP dataset is smaller than SILVA database but more selective. The website tool is limited to 2,000 sequences.

### Conclusions/Significance

Hypervariable region tag sequencing using either the V3 or the V6 region, and presumably other hypervariable regions, is an effective means for assigning taxonomy and provides great advantages over traditional sampling. A tag mapping process such as GAST with an extensive database of rRNA genes such as our RefSSU derived from SILVA can map tag sequences to the same taxonomy as their source genes at better than a 99% correlation rate for commonly studied environments such as the human microbiome and better than 96% for less commonly studied environments such as deep-sea vents. The V3 and V6 regions have only minimal ambiguity in mapping to the SSU rRNA gene all the way to the genus level. While tags can map to more than one SSU rRNA source, these cross-mappings between taxa are infrequent and do not compromise the overall methodology. We show that these short hypervariable region tags contain adequate information to uniquely and accurately map the phylogeny with a 98% or greater fidelity even without an exact match in the reference database or with potential multiple copies in the database. GAST is accurate for tags as much as 15% divergent from their nearest reference match, although there were very few tags that far from the current set of reference SSU rRNA sequences. The GAST distance, like BLAST scores or e-values, should be maintained and used as an assessment for the likely reliability of the GAST assignment of more divergent tags. The consistently high correspondence of the hypervariable region tags vs. long SSU rRNA taxonomies shows the robustness of the GAST process and the use of tags as surrogates for the full-length rRNA genes even for microbial environments that are not well-represented in the reference databases.

Massively-parallel pyrosequencing of tags can be used to great advantage over traditional sequencing of full-length rRNA genes to explore both the diversity and relative abundance of microbial populations. Further research into hypervariable region tag sequencing may uncover advantages of one region over another, such as the relative levels of microvariation, length of sequence, density of homopolymers (which can lead to pyrosequencing errors), ability to identify to the species level, or the merits of different amplification primers. Tag sequencing yields similar taxonomy and relative abundance values as conventional sequencing of full-length SSU rRNA genes, but provides more reads, uncovers more organisms, avoids assembly, and costs less per read than conventional sequencing of full-length SSU rRNA genes. As the technology continues to improve, yielding greater read counts and longer sequences, pyrosequencing will provide even greater opportunities for tag sequencing, such as the use of longer hypervariable regions or combinations of variable regions, and ever-greater sampling depth. This process will also improve as reference databases of SSU rRNA genes continue to grow. The great advantage of hypervariable region tag sequencing is that it can take advantage of massively-parallel pyrosequencing, sampling to depths several orders of magnitude greater than previously achieved, facilitating the exploration of the vast diversity of microbial populations and the rare biosphere.

## Materials and Methods

### Creating the Reference Database of Full-Length, V3, and V6 SSU rRNA Sequences

We downloaded 503,971 aligned small subunit rRNA sequences from the SILVA database, version 92 [35]. Using the SILVA quality assessments, we eliminated low-quality sequences (sequence quality < = 50, alignment quality < = 50, pintail score < = 40). SSU rRNA genes whose sequences were identical were flagged as redundant. The resultant dataset included 417,433 unique sequences, of which 99% were between 350 and 2000 nt in length. Although the sequences vary in length and coverage of the full-length SSU rRNA gene, we refer to these sequences as “long” or “full-length” sequences for the purposes of this paper, and the dataset of these sequences as RefSSU. From all aligned RefSSU sequences, we extracted the V3 and V6 hypervariable regions, defined as homologous positions between positions 338 and 533 of the *E. coli* SSU rRNA sequence (U00096) for V3, and 967 to 1046 for V6. Sequences shorter than 50 nt or containing ambiguous bases were culled. We removed all gap characters to create a set of 293,265 V3 reference tags (RefV3 database) and 195,344 V6 reference tags (RefV6 database). The higher representation of sequences spanning the V3 region in molecular databases is likely a consequence of the experimental design used to generate PCR amplicon libraries favoring the beginning of the molecule. These databases include 123,206 unique V3 tag sequences and 59,830 unique V6 tag sequences. Most V3 sequences (99+%) range in length from 80 nt to 180 nt (max 447), while the most V6 sequences (99+%) range from 50 nt to 80 nt with a maximum of 349 nt (http://vamps.mbl.edu/resources/databases.php).

We classified all bacterial and archaeal long sequences directly with the Ribosomal Database Project Classifier (RDP) [28]. We used only RDP classifications with a bootstrap value of > = 80%. If the bootstrap value was <80%, the taxonomic assignment was moved to a higher classification level until an 80% or better bootstrap value was achieved. For example, if the genus assignment had a bootstrap value of 70%, but the family had a value of 85%, that sequence would be assigned only as far as family and not to genus. RDP Classifier does not classify sequences below the genus level but the GAST process is not inherently limited to genus; its resolution is constrained by the taxonomy of the reference sequence database. The accuracy of GAST will improve in response to refinements of the reference database including increased number of taxonomically-resolved sequences, removal of cryptic chimeric and short sequences, improvement of taxonomic identities for long sequences, and elimination of low quality entries.

### Sampling of Human Gut Microbiome

Detailed methods are described in the companion paper [30]. Briefly, we extracted DNA from fecal samples of three individuals before, during, and after a 5-day course of the antibiotic ciprofloxacin, then performed PCR with primers designed to amplify the SSU rRNA gene (hereafter referred to as “full-length”), as well as the V3 and V6 hypervariable regions of the full-length gene. The forward primers for the full-length product were 90% bacterial primer 8F (AGAGTTTGATCMTGGCTCAG) and 10% 8F-Bif targeting *Bifidobacteria* (AGGGTTCGATTCTGGCTCAG), and were paired with the 3 domain reverse primer 1391R (GACGGGCGGTGTGTRCA). Re-conditioning PCR reactions were performed for the full-length gene amplifications. Full-length amplicons were cloned and sequenced with Sanger dideoxy methods, assembled with phrap [36], aligned with NAST [31] and evaluated for chimeras with Bellerophon [37] (GenBank Accession numbers: EU761594-EU768801). The V3 and V6 amplicon libraries were sequenced with a Roche GS FLX pyrosequencer.

The primers spanning V3 were 338F (ACT CCT ACG GGA GGC AGC AG) and 533R (TTA CCG CGG CTG CTG GCA C). The primers spanning V6 were a combination of 967F primers (CAA CGC GAA GAA CCT TAC C and ATA CGC GA[AG] GAA CCT TAC C) and a combination of 1046R primers (AGG TGN TGC ATG GCT GTC G and AGG TGN TGC ATG GTT GTC G).

### Generation of Deep-Sea Vent Microbe Long Sequences

Detailed methods are described elsewhere [15]. Briefly, DNA was extracted from deep-sea vent samples and PCR was conducted using primers designed to amplify a 1000 bp region of SSU rRNA. The amplicons were sequenced bidirectionally using primers T3 (5′- ATT AAC CCT CAC TAA AGG GA) and T7 (5′- TAA TAC GAC TCA CTA TAG GG). Sequencing was performed on an Applied Biosystems 3730XL capillary sequencer (GenBank Accession numbers DQ919170-DQ910173).

### Generation of In Silico Hypervariable Region Tag Subsequences from Full-Length Sequences

We aligned each dataset of full-length sequences with MUSCLE [38] (default parameters). We located the position of the V6 primers (967F and 1046R), and the V3 primers (338F and 533R) in each alignment and extracted, in silico, the hypervariable regions from each of the aligned full-length sequences.

### Generation of V3 and V6 Tag Sequences

The V3 and V6 amplicon libraries were sequenced on a Roche Genome Sequencer GS-FLX using standard protocol. The binary sff files were converted to text using default parameters of sffinfo (Newbler distribution-Software Release: 1.1.03.24). The data were imported into a MySQL 5 database along with metadata describing the run. We filtered for high-quality sequences by removing from the dataset any sequence that did not have an exact match to the proximal primer, contained fewer than 50 bases, or had one or more ambiguous bases (Ns) within the sequence as per Huse et al [39]. We used these quality filters preferentially over the GS-FLX quality scores, which are based on homopolymeric sequences. We removed the exact proximal primer using exact string matching. Since the GS-FLX technology at the time of sequencing was restricted to reads shorter than the maximum hypervariable region length, we could not require the presence of a perfect distal primer for all tag sequences. We used a combination of BLAST (-W 7 –q -1 –E 2 –G 1 –S 1) and the EMBOSS program fuzznuc [40] (-pmismatch 3) to locate and remove inexact matches to the distal primer.

### Assigning Taxonomic Classification to Hypervariable Region Tags through GAST

In Sogin et al. [9], we proposed a tag mapping methodology, GAST (Global Alignment for Sequence Taxonomy) to assign a taxonomic classification to environmental V6 tags (http://vamps.mbl.edu/resources/software.php). The first step in GAST is to BLAST each tag against the RefV3 or RefV6 database (no minimum score, expectation value or other cutoffs were imposed). Because the top BLAST hit may not have the highest overall similarity to the tag sequence, particularly because edge-effects in such a short region can be pronounced, we aligned the tag sequence to the reference hypervariable region tags corresponding to the top 100 BLAST hits. We used MUSCLE [38] (with parameters –diags and -maxiters 2 to reduce processing time) because it is well suited to high-throughput experiments. We calculated the global distance from the sample tag to each of the aligned reference sequence tags as the number of insertions, deletions and mismatches divided by the length of the tag, using quickdist [9]. We considered the reference sequence or sequences with the minimum global distance to be the top GAST match(es). The top BLAST hit was frequently the best global match; however, for 5% to 25% of tags the best global match was to a reference sequence with a lower BLAST score.

For each tag, we identified all of the reference long sequences in RefSSU that contained the exact hypervariable sequence of the top GAST match(es). We compared the taxonomic classification of all corresponding SSU rRNA sequences (with RDP bootstrap values> = 80) and generated a consensus taxonomy. If two-thirds or more of the full-length sequences shared the same assigned genus, the tag was assigned to that genus. If there was no such agreement, we proceeded up one level to family. If there was a two-thirds or better consensus at the family level, we assigned this taxonomy to the tag, and if not, we continued to proceed up the tree. Occasionally, a tag could not be assigned taxonomic classification at the domain level. This was because the RDP Classifier could not assign a domain with an adequate bootstrap value, rather than a tag mapping to full-length sequences from different domains. These may represent novel organisms whose taxonomy has not yet been determined. Sample tags that did not have a single BLAST match in the RefSSU database also were not given a taxonomic assignment. We chose to use a 66% (two-thirds) majority although other values or a distributional vs. strict percentage approach can be implemented. We reviewed nearly 17 million tags in our sequencing database (primarily of the V6 region) from a wide range of studies using the 66% majority as the threshold for assignment. A distribution curve of voting majority did not show any obvious break points (graph not shown), although 95% of the tags had a voting majority of 75% or better, and 90% had a voting majority > = 83%.
