# Correction: Exploring Microbial Diversity and Taxonomy Using SSU rRNA Hypervariable Tag Sequencing

**DOI:** 10.1371/annotation/3d8a6578-ce56-45aa-bc71-05078355b851

**Published:** 2008-12-03

**Authors:** Susan M. Huse, Les Dethlefsen, Julie A. Huber, David Mark Welch, David A. Relman, Mitchell L. Sogin

The fourth author's name was listed incorrectly in the article's citation. The correct citation is: Huse SM, Dethlefsen L, Huber JA, Mark Welch D, Relman DA, et al. (2008) Exploring Microbial Diversity and Taxonomy Using SSU rRNA Hypervariable Tag Sequencing. PLoS Genet 4(11): e1000255. doi:10.1371/journal.pgen.1000255

